# Neuron-specific enolase and Tau protein as biomarkers for sepsis-associated delirium: a cross-sectional pilot study

**DOI:** 10.31744/einstein_journal/2025AO1244

**Published:** 2025-03-28

**Authors:** Agnes Araújo Sardinha Pinto, Maira Mello de Carvalho, Juliana Bahia Santos, Rebeca Souza da Silva, Hermes Vieira Barbeiro, Luz Marina Gómez Gómez, Ian Ward Abdalla Maia, Júlio Flávio Meirelles Marchini, Flávia Barreto Garcez, Thiago Junqueira Avelino-Silva, Lucas de Moraes Soler, Matheus Menão Mochetti, Heraldo Possolo de Souza, Júlio Cesar Garcia Alencar

**Affiliations:** 1 Discipline of Clinical Emergencies Faculdade de Medicina Universidade de São Paulo São Paulo SP Brazil Discipline of Clinical Emergencies, Faculdade de Medicina, Universidade de São Paulo, São Paulo, SP, Brazil.; 2 Faculdade de Medicina de Bauru Universidade de São Paulo Bauru SP Brazil Faculdade de Medicina de Bauru, Universidade de São Paulo, Bauru, SP, Brazil.; 3 Department of Medicine Hospital Universitário Universidade Federal de Sergipe São Cristovão SE Brazil Department of Medicine, Hospital Universitário, Universidade Federal de Sergipe, São Cristovão, SE, Brazil.; 4 Hospital das Clínicas Faculdade de Medicina Universidade de São Paulo São Paulo SP Brazil Laboratório de Investigação Médica em Envelhecimento, Serviço de Geriatria, Hospital das Clínicas, Faculdade de Medicina, Universidade de São Paulo, São Paulo, SP, Brazil.; 5 Universidade Estadual de São Paulo “Julio de Mesquita Filho” Botucatu SP Brazil Universidade Estadual de São Paulo “Julio de Mesquita Filho”, Botucatu, SP, Brazil.

**Keywords:** Biomarkers, Delirium, Emergency medicine, Neuroinflammatory diseases, Sepsis

## Abstract

**Objective:**

Sepsis-associated delirium is a common cerebral manifestation in patients with sepsis, potentially caused by a combination of neuroinflammation and other neurophysiological disorders. This study investigated the expression of neuron-specific enolase and Tau protein as biomarkers in patients with sepsis-associated delirium. While neuron-specific enolase and Tau protein are known to be associated with brain injury, their diagnostic potential in patients with sepsis-associated delirium is not well understood.

**Methods:**

This cross-sectional pilot study evaluated plasma levels of neuron-specific enolase and Tau protein in patients with delirium and sepsis to explore their potential for identifying sepsis in patients admitted to the emergency department.

**Results:**

A total of 25 patients with delirium were analyzed, 56% of whom had sepsis. Patients with sepsis exhibited significantly higher neuron-specific enolase levels (2.7ng/mL [95%CI= 2.2-3.2] *versus* 1.3 ng/mL [95%CI= 0.8-2.5], p<0.003) and Tau protein levels (96.1pg/mL [95%CI= 77.0-111.3] *versus* 43.0pg/mL [95%CI= 31.2-84.5], p<0.003) compared to patients without sepsis. Neuron-specific enolase and Tau protein thresholds of >2.08ng/mL and >59.27pg/mL, respectively, demonstrated 90% specificity for identifying sepsis in patients.

**Conclusion:**

Neuron-specific enolase and Tau protein levels were significantly higher in patients with sepsis than in those without, underscoring their potential ability to identify the infectious etiology of delirium in older patients admitted to emergency departments. **Clinical Trials** #RBR-233bct.

## In Brief

In this study, Pinto et al. identified significantly higher levels of neuron-specific enolase and Tau protein in older patients with sepsis-associated delirium in the emergency department, suggesting the potential of these biomarkers as diagnostic tools in this population.

## Highlights

■ Biomarkers of brain injury, such as neuron-specific enolase and Tau proteins, are higher in older patients with sepsis and delirium.

■ Diagnosing sepsis in patients with delirium can be challenging.

■ Early identification of sepsis is key to managing sepsisassociated delirium.

This manuscript has not been published previously and is not under consideration for publication elsewhere, either in whole or in part, in any language.

## Graphical abstract

**Figure:**
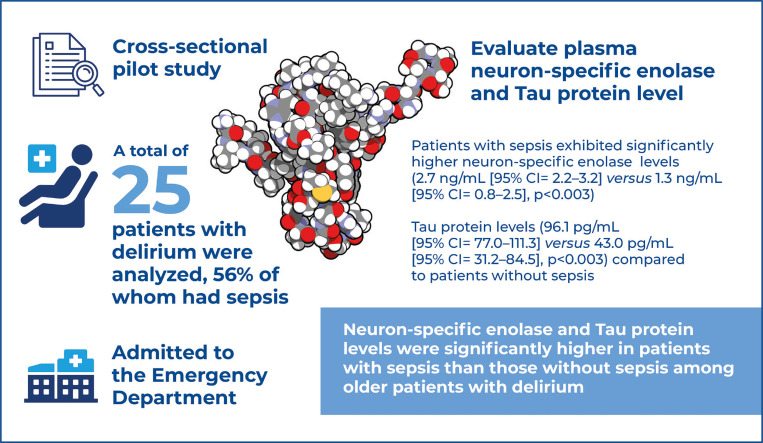

## INTRODUCTION

Delirium is defined by the acute onset of fluctuating levels of consciousness, impaired attention, disorganized thinking, and cognitive dysfunction.^1^ It is frequently observed in emergency departments (EDs) and is associated with longer hospital stays, higher mortality rates, increased admissions to intensive care units (ICUs), and long-term cognitive impairment.^2,3^

Various conditions, including metabolic disorders, intoxication, prolonged immobilization, and, most commonly, infections, can trigger delirium in susceptible patients.^4^ However, because there are no definitive blood or imaging tests to diagnose delirium or identify its underlying cause, patients often use multiple invasive procedures to determine the etiology and guide appropriate treatment.^5^

The most widely accepted theory of delirium pathophysiology involves systemic injury and inflammation, leading to the blood-brain barrier (BBB) disruption, glial and neuronal activation, increased inflammation, and subsequent cell death.^6^ Similarly, sepsis can cause endothelial and BBB damage, resulting in the release of neuronal proteins into the bloodstream.^7^ Neuron-specific enolase (NSE) is a recognized biomarker of neuronal death and has prognostic value in patients who are neurocritical. Tau protein, which plays a key role in stabilizing axonal microtubules, has been linked to cognitive impairment. Both NSE and Tau are serum biomarkers indicative of brain injury and have been associated with delirium and, more recently, sepsis.^8,9^

## OBJECTIVE

This study evaluated the potential correlation between plasma levels of brain cell injury biomarkers-neuron-specific enolase and Tau protein- and delirium in patients. Additionally, it sought to identify sepsis as a potential cause of delirium in patients admitted to emergency departments.

## METHODS

### Study design, setting, and population

This cross-sectional pilot study analyzed patients aged 65 years or older with acute medical emergencies admitted to the ED of a tertiary university hospital (*Hospital das Clínicas*) in São Paulo, Brazil, between September 30, 2019, and March 17, 2020. *Hospital das Clínicas* is a 2,200-bed facility dedicated to caring for high-complexity medical and surgical patients. This study is part of an ongoing research project on Biomarkers and Clinical Scores in Critically Ill Patients, which investigates serum biomarkers in older patients in the ED and their outcomes.^10^

**Figure 1:**
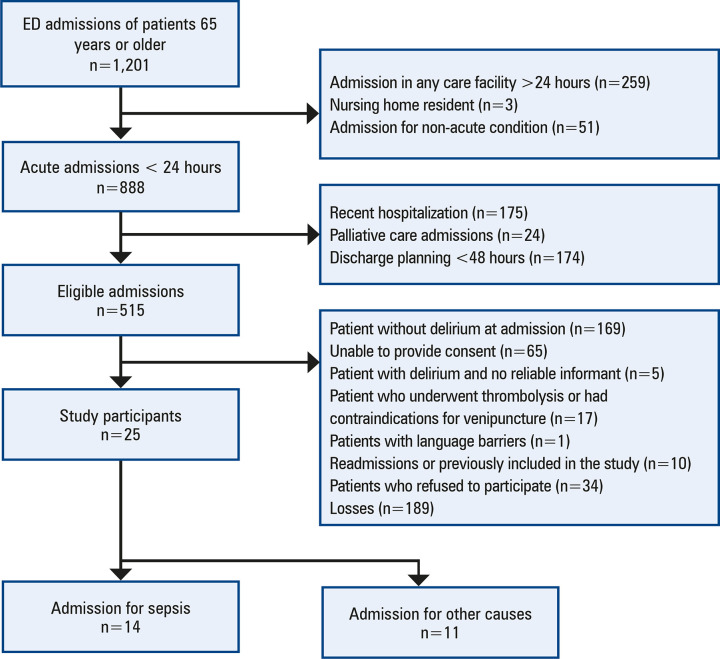
Flowchart for the selection of patients

We evaluated all older patients admitted with delirium for inclusion in this study. Patients were excluded if they met any of the following criteria: hospitalization for more than 24 h in any healthcare facility, hospitalization within 30 days before admission, anticipated hospital discharge in less than 48 h, or receipt of end-of-life care. Additional exclusions included patients without delirium at admission, those unable to provide consent, delirious patients without a reliable informant, patients who had undergone thrombolysis or had contraindications for venipuncture, those facing significant language barriers, readmissions, previously included patients, those who refused participation, and those lost to follow-up (Figure 1).

The study protocol was approved by the Ethics Committee for the Analysis of Research Project (CAPPesp) of the *Hospital das Clinicas, Faculdade de Medicina, Universidade de São Paulo* (CAAE: 77169716.2.0000.0068; # 2.327.363). Written informed consent was obtained from all participants. We report our results in accordance with the Strengthening the Reporting of Observational Studies in Epidemiology (STROBE) guidelines.^(11^

### Delirium assessment

We used the Confusion Assessment Method (CAM) algorithm for admission assessment.^12^ Our standardized interview protocol included a brief neuropsychiatric history, cognitive screening (10-CS), attention testing (reciting the days of the week backward and the vigilance A test), level of consciousness assessment (Richmond Agitation and Sedation Scale [RASS]), and reviews of the electronic medical records.^13-16^

Raters underwent training sessions before the study began, which involved simulations and bedside evaluations. We achieved high inter-rater reliability for diagnosing CAM-based delirium (>95%). In cases where raters were unsure about the presence of delirium, two experienced physicians re-evaluated the assessments to confirm the final diagnosis.

### Brain injury biomarker measurements

Sampling procedures were performed after patient inclusion. Three registered nurses collected 30ml of blood via venipuncture. Blood samples for brain injury biomarker analyses were immediately centrifuged for 10 min. Plasma was preserved at -20°C for up to 48 h before being transferred to a -80°C freezer for long-term storage and further processing.

We used the Milliplex^®^ magnetic bead immunoassay and the MAGPIX^®^ System (Merck Millipore, USA) to measure cytokine plasma levels.

### Statistical analyses

Our primary outcome was the diagnosis of sepsis at admission, based on the Sepsis-3 criteria, which included the presence of a suspected or confirmed infection and a change of two or more points in the Sequential Organ Failure Assessment (SOFA) score from baseline. Sepsis was confirmed through a complete review of medical records, including a detailed evaluation of clinical symptoms, medical history, physical examination findings, and laboratory results such as blood cultures and other relevant tests. Although radiological data were not directly used for sepsis diagnosis, they were reviewed for additional context regarding infection-related findings, and microbiological data were considered if available. The primary focus was on clinical and laboratory evidence consistent with the Sepsis-3 criteria.^17^ All enrolled patients were included in the analysis on an intention-to-treat basis.

The number of participants was determined based on feasibility, available resources, research staff capacity, and the number of eligible patients, which aligned with current recommendations.^18^ Despite our ED providing medical care to 800 older patients monthly, with 30% being eligible for hospitalization, most had been transferred from less complex hospitals and hospitalized for more than 24 h at the time of recruitment. Recruitment was completed one month ahead of schedule due to the onset of the COVID-19 pandemic.

We used the Shapiro-Wilk test to assess the normality of variable distributions. Sepsis occurrence was analyzed using unpaired *t-tests* or Mann-Whitney U tests, depending on the normality of variable distributions. Categorical variables were analyzed using Pearson’s χ^2^ test. All analyses were performed using Stata software (StataCorp, 2021, Stata Statistical Software: Release 17*,* College Station, TX: StataCorp LLC).

## RESULTS

### Patient demographic and clinical characteristics

We analyzed 1,201 patients, of whom 25 (2.1%) were older patients diagnosed with delirium at admission. The cohort was predominantly female (n=14, 56%), with a mean age of 78.2 (± 8.0) years. Patients were hospitalized for a median of 8 (4-15) days, and 9 (36%) died (Figure 1, Table 1).

**Table 1:** Characteristics of the patients

	Septic patients (n=14)	Non-septic patients (n=11)	p value
Age, median (IQR), years	76 (12.3)	79 (18)	0.3*
Female, n (%)	6 (42.8)	8 (72.7)	0.1^‡^
Education level, median (IQR), years	8 (5.2)	3 (4)	0.007*
Charlson Comorbidity Index, median (IQR)	2 (2.2)	3 (2)	0.9*
Polypharmacy, n (%)	8 (57.1)	4 (36.3)	0.3^‡^
Katz Index, median (IQR)	4.5 (6)	4 (11)	0.6*
Frail, n (%)	6 (42.8)	5 (45.4)	0.9^‡^
Dementia, n (%)	2 (14.2)	1 (9.0)	0.7^‡^
ICU admission, n (%)	4 (28.5)	4 (36.3)	0.7^‡^
In-hospital mortality, n (%)	4 (28.5)	5 (45.4)	0.4^‡^

Variables are expressed as absolute numbers (%), except for age, education level, and Charlson Comorbidity Index, which are expressed as medians (interquartile range [IQR]).

*p values were calculated using the nonparametric Mann-Whitney test for continuous quantitative variables; ^‡^ p values were calculated using the χ^2^ test for categorical variables.

ICU: intensive care unit.

Among the patients with delirium, 14 (56%) were diagnosed with sepsis. The most common site of infection in these patients with sepsis was pneumonia (n=6, 42.8%), followed by skin and soft tissue infections (n=3, 21.4%), urinary tract infections (n=2, 14.2%), and abdominal infections (n=2, 14.2%). Patients without sepsis had conditions including stroke (n=4, 36.3%), acute heart failure (n=2, 18.1%), and acute upper gastrointestinal bleeding (n=2, 18.1%).

### Main results

**Figure 2:**
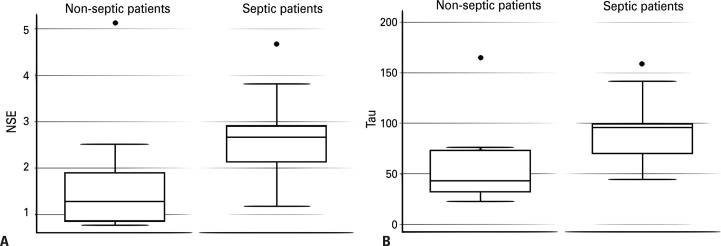
(A) Neuron-specific enolase in patients with and without sepsis and (B) Tau protein in patients with and without sepsis

Patients with sepsis had significantly higher NSE and Tau protein levels than those without sepsis (Table 2, 3 and Figure 2).

**Table 2:** Characteristics of biomarkers

	Septic patients (n=14)	Non-septic patients (n=11)	p value*
NSE, median (IQR), [95% CI]	2.7 (0.9), [2.2–3.2]	1.3 (1.0), [0.8–2.5]	0.003
Tau, median (IQR), [95% CI]	96.1 (30.1), [77.0–111.3]	43.0 (41.6), [31.2–84.5]	0.003

* p values were calculated using the nonparametric Mann-Whitney U test.

**Table 3:** Characteristics of the neuron-specific enolase and Tau protein

	Value	Sensitivity %	Specificity %	LR +	LR -	AUC
NSE	2.08	85.7	81.2	4.7	0.17	0.84
Tau	59.27	92.9	72.7	3.4	0.09	0.84

NSE: neuron-specific enolase; LR: likelihood ratio.

## DISCUSSION

This study analyzed older patients with delirium and found that plasma levels of NSE and Tau protein were higher in those with sepsis. Sepsis is a potentially fatal condition caused by a dysregulated systemic response to infection,^17^ with the brain being particularly vulnerable to its effects. Symptoms range from mild confusion to deep coma.^19^ A common neurological complication is sepsis-associated delirium (SAD), which is thought to result from a combination of neuroinflammation, disturbances in cerebral perfusion, BBB dysfunction, and altered neurotransmission.^20^

Effective management of SAD depends on the early detection and treatment of underlying sepsis, as controlling SAD after it develops is challenging.^20^ Delirium may be the only clinical manifestation of sepsis in older patients, and diagnosing it can be delayed due to the need to investigate its etiology.^21^ However, the utility of biomarkers, neuroimaging, and electroencephalograms for diagnosing SAD remains controversial.^22^

Plasma levels of NSE and Tau protein have been associated with brain injury and delirium.^23,24^ Neuron-specific enolase is a promising peripheral blood marker of brain injury as it is a cytosolic enzyme primarily expressed in neurons and neuroendocrine cells, with a high concentration in the brain.^25^ Neuron-specific enolase has demonstrated value in assessing the severity of brain injury and early prognosis following traumatic brain injury,^26^ cardiac arrest,^27^ and sepsis.^28^

Tau is a microtubule-associated protein highly expressed in neurons, which regulates axonal microtubule stability. Its levels increase in response to various neurodegenerative conditions and following stroke.^29,30^ Elevated Tau protein levels can also result from injury, synaptic activity, or synaptic pruning, making their interpretation complex.^31^ Plasma Tau protein levels have been linked to the incidence and severity of postoperative delirium.^24^ To our knowledge, this study is the first to demonstrate an association between Tau protein levels and SAD.

This study has some limitations. First, while NSE and Tau protein levels were measured in peripheral blood, these values may not fully correspond to those in the brain. However, this was an ED-based study designed to be feasible and reproducible.

Second, many patients were not enrolled during the study period. Recruitment occurred daily in the morning, but some patients were transferred to wards or ICUs within hours of ED admission. Additional factors, such as difficulties obtaining consent, loss of follow-up, and logistical challenges, further contributed to patient losses. To address these issues, we implemented measures to mitigate patient attrition. These included enhanced follow-up efforts through multiple phone and mail attempts to track and retain participants. We also reviewed and simplified the consent procedures to better accommodate patient needs, along with providing additional support when required.

Finally, although our findings suggest that plasma NSE and Tau protein changes are associated with sepsis in patients with delirium, the underlying mechanisms remain unclear. These changes could result from endothelial dysfunction, neuronal injury, synaptic alterations, or a combination of these processes.

## CONCLUSION

Neuron-specific enolase and Tau protein levels are established biomarkers of brain injury. In this study, among the older patients with delirium, these biomarker levels were higher in those with sepsis patients compared to those without sepsis. We propose that plasma levels of neuron-specific enolase and Tau protein be further investigated as potential tools for identifying the infectious etiology of delirium in older patients in emergency departments.

AVAILABILITY OF DATA AND MATERIAL

Data supporting the findings of this study are available from the corresponding author upon request.
